# Past, Present, and Future Perspectives on Computer-Aided Drug Design Methodologies

**DOI:** 10.3390/molecules28093906

**Published:** 2023-05-05

**Authors:** Davide Bassani, Stefano Moro

**Affiliations:** 1 Pharmaceutical Research & Early Development, Roche Innovation Center Basel, F. Hoffmann—La Roche Ltd., 4070 Basel, Switzerland; davide.bassani@roche.com; 2 Molecular Modeling Section (MMS), Department of Pharmaceutical and Pharmacological Sciences, University of Padova, Via Marzolo 5, 35131 Padova, Italy

**Keywords:** CADD, computational, chemistry, drug, design, AI, molecular docking, molecular dynamics, learning

## Abstract

The application of computational approaches in drug discovery has been consolidated in the last decades. These families of techniques are usually grouped under the common name of “computer-aided drug design” (CADD), and they now constitute one of the pillars in the pharmaceutical discovery pipelines in many academic and industrial environments. Their implementation has been demonstrated to tremendously improve the speed of the early discovery steps, allowing for the proficient and rational choice of proper compounds for a desired therapeutic need among the extreme vastness of the drug-like chemical space. Moreover, the application of CADD approaches allows the rationalization of biochemical and interactive processes of pharmaceutical interest at the molecular level. Because of this, computational tools are now extensively used also in the field of rational 3D design and optimization of chemical entities starting from the structural information of the targets, which can be experimentally resolved or can also be obtained with other computer-based techniques. In this work, we revised the state-of-the-art computer-aided drug design methods, focusing on their application in different scenarios of pharmaceutical and biological interest, not only highlighting their great potential and their benefits, but also discussing their actual limitations and eventual weaknesses. This work can be considered a brief overview of computational methods for drug discovery.

## 1. Introduction: The Benefits of Computational Methods for Drug Discovery

### 1.1. The Drug Discovery Pipeline and the Problem of Candidate Selection

The drug discovery process is an extremely money- and time-consuming procedure, which is necessary to guarantee the safety and the quality of novel therapeutical entities entering the market. It has been reported that a single novel small molecule can require up to 14 years and more than one billion dollars in the several steps from target assessment to regulatory approval [1,2]. Moreover, the failure risk in the pharmaceutical scenario is known to be one of the highest in the industry. Indeed, it has been estimated that just one or two out of 10,000 screened molecules can effectively become drugs [3].

Another major challenge in the medicinal field is the tremendously extended chemical space forming the “drug-like” environment. It has been calculated that the number of small molecules included in such a concept would be roughly 10^60^ [4], which is a number higher than the seconds of life in the entire Universe. Such a vast chemical space is unfeasible to explore, and this is even more true from an experimental perspective. Indeed, even if the most technological high-throughput screening (HTS) methods today can evaluate the on-target activity of hundreds of thousands of compounds/week [5], their capacity would never reach the order of magnitude of the potential candidates for that specific biological entity. This limitation can be overcome by medicinal chemists by bringing the candidate selection problem from a laboratory setup to a “virtual environment”. Specifically, one of the first ideas that came up was to exploit computers to perform molecular “virtual” screenings before the experimental ones. This approach, which was called “high-throughput virtual screening” (HTVS), still represents one of the main applications of computational methodologies in drug discovery [6]. Indeed, the capacity of the virtual screening depends essentially on the computer power of the infrastructure exploited for the purpose, and it is much faster and cheaper than the preparation and execution of experimental assays. As a demonstration of this, with the actual combinations of software and hardware, the evaluation of several millions of compounds/day is achievable [7,8,9] (even billions, as stated by the researchers at the Oak Ridge National Laboratory working on the SUMMIT supercomputer [10], which was recently exploited for an ultralarge GPU-accelerated virtual screening against SARS-CoV-2 main protease [11]). In recent decades, many academic and industrial groups have extensively put efforts into the improvement of these methods, making them among of the pillars in the current drug discovery pipeline, especially in the early discovery phases. 

### 1.2. The Application of Computational Methods in Drug Discovery

To go a little bit more into detail, the drug discovery process can be divided into five main steps [12], according to the United States Food and Drug Administration (US FDA). The first is the “discovery and development” phase, which includes hit identification, hit-to-lead (H2L), and lead optimization. The first of these consists of not only highlighting some molecular candidates with a good activity profile against the desired target, but also presenting pharmacokinetic (PK) or pharmacodynamics (PD) limitations. Talking in terms of on-target potency, the hit compounds are usually in the micromolar (μM) range of activity, and they are tendentially not so selective. Even with all these pharmaceutical problems, the hit compounds are very important to give some hints to the drug design teams, being very useful starting points for further modification [13,14].

The second passage consists of the hit-to-lead optimization phase. In this step, the hit compounds are modified with different methodologies to improve their on-target activity and selectivity, always keeping their PK/PD profile under strict evaluation [15]. After this process, the optimized molecular candidates take the name of “lead” compounds and are usually very active (in the nanomolar range for what concerns potency) and reasonably selective. These compounds then enter the second main step, which is the preclinical experimental phase, where they are tested in animal and organoid models to assess their safety and efficacy. The third phase, which is also the longest, consists of human clinical trials. These are divided into three main sections (I, II, and III), each with a different endpoint and with increasing patients enrolled in the tests. Just after a positive outcome of the therapy with the new candidate in the clinical phase III, the commercialization of the drug can be asked the regulatory agencies (e.g., EMEA for Europe and FDA for the USA), determining the opening of the fourth phase of drug development. After this, the fifth and last step consists of post-market drug safety monitoring.

Even if the preclinical and clinical trials constitute the longest and most expensive part of the drug discovery pipeline, not so much can be implemented to reduce them, mainly because of the extremely delicate outcomes in terms of safety and efficacy that they provide. On the other hand, the steps that lead from the hits up to the lead compoundscan be extensively optimized, and that is the space in which computational design approaches have found their main application. Indeed, other than exponentially improving the number of virtual compounds that can be evaluated daily, these methodologies offer also the possibility to deeply analyze the patterns in the chemical data under evaluation; furthermore, they make the rational design of such entities much more than achievable [16]. Indeed, the advances in spectroscopic techniques, together with the tremendous improvement in computer graphics, allowed the visual inspection of proteins, ligands, and biologically relevant complexes in the routines of drug design groups [17]. With such computational approaches, it is possible to effectively design new molecular candidates; for this reason, the techniques of this family have been gathered under the common name of “computer-aided drug design” (CADD).

### 1.3. The Main Methodology Branches in CADD

**Figure 1:**
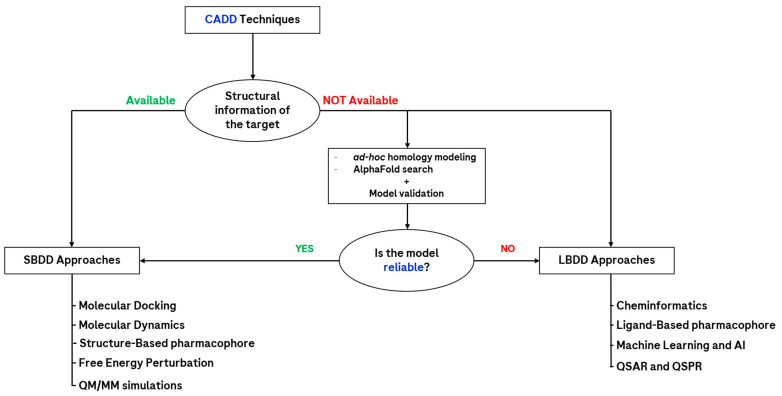
Scheme representing the main computational approaches available to the CADD scientist in drug discovery. As can be seen, a key factor is represented by the availability of structural information about the target. Abbreviations: QM/MM = quantum mechanics/molecular mechanics; AI = artificial intelligence; QSAR = quantitative structure–activity relationship; QSPR = quantitative structure–property relationship.

How a computational chemist approaches a pharmacological problem can be multifaceted, but the main underlying factor discriminating the procedure is the quantity of data available on the topic examined (Figure 1). Indeed, one of the most important determinants is the presence of experimental structural information on the target of interest [16], which can currently be obtained using various techniques, among which the most relevant are certainly nuclear magnetic resonance (NMR), X-ray crystallography (XR), and cryogenic electron microscopy (cryo-EM) [18]. If such data are available, then the approach chosen by the scientist usually addresses the application of an ensemble of computational methods, which take advantage of this, such as molecular docking and molecular dynamics. Because of this, those techniques fall under the family of the “structure-based drug design” (SBDD) approaches. On the other hand, when no experimental information about the target three-dimensional structure is available, then two main possibilities are open to CADD scientists. The first consists of searching for close homologs of the target of interest to create a computational model of it (also known as the *homology model*), which would then be tested for structural reliability and used with SBDD techniques [19]. In the latest years, a huge revolution in the field of protein structure prediction was represented by the creation of AlphaFold [20], which is now also available in version 2.0. This algorithm, developed by the company DeepMind, exploits artificial intelligence (AI) approaches to predict the three-dimensional structure of a biological entity on the basis of its sequence, which is also associated with a confidence score in its different functional regions. Another limitation of this method, which still necessitates the homology models created ad hoc by the scientists, is represented by the fact that it predicts only one conformational state (usually the inactive one) of the targets of interest (this was partially resolved with the recent implementation of AphaFill [21]), and that not all the proteins are still included in the AlphaFold database (e.g., many viral proteins are still lacking) [22].

Another main option for the CADD scientist lacking the structural information of the biological entity of interest is to exploit just the information coming from the ligands which have been tested on it, extrapolating from them enough information to build reliable quantitative structure-activity relationship (QSAR) models [23]. These approaches were among the first used in rational drug design and now fall into the category of the “ligand-based drug design” (LBDD) techniques. This family comprises methods such as pharmacophore search [24] and matched molecular pair analysis [25]; even if their application over the years has lowered, giving more and more space to the SBDD techniques, they are still widely used in several scenarios. Lastly, the latest advances in computer science, together with the exponential increase in the application of machine learning (ML) and artificial intelligence approaches, provided another powerful instrument to CADD scientists [26]. Indeed, when huge amounts of data are available for a defined context (regarding both the target and the ligands), these approaches can be proficiently used for the proper prediction of molecular properties of pharmaceutical relevance. Moreover, the creation and maintenance of an “intelligent” algorithm in the most recent years have allowed such “computational brains” to create novel chemical structures, through an approach known as de novo drug design [27].

## 2. Discussion

### 2.1. Ligand-Based Drug Design (LBDD)

By far the most used approaches in the dawn of rational drug design, these techniques rely only on the structural information of the molecular structures tested on the desired target. The main goal of such methods is to identify patterns in the data that can be extrapolated to guide the further steps to take in terms of drug discovery. Those patterns are usually identified as “quantitative structure–activity relationship” (QSAR) models and should allow scientists to obtain a discrete and quantitative correlation between chemical moieties and pharmacological outcomes. Some of the techniques that are mostly used for ligand-based determination of these interconnections are cheminformatics [28] (e.g., matched molecular pair analysis), ligand-based pharmacophore search, and Free–Wilson analysis [29]. Some very famous equations, which are fundamental for the birth and the rise of QSAR modeling, were advanced by Hansch, Hammet, and Taft [30]. Even if some of the cited methods are still widely used, their main disadvantage remains related to the low level of generalization that they can provide. Indeed, they tend to work only on highly congeneric series of ligands; in other cases, they require great amounts of experimental data to provide reliable results. Moreover, these approaches do not take into consideration the conformational freedom of the ligands, focusing only on the 2D representation of the molecules considered. Together with the rising importance of structure-based approaches, and of “three-dimensionality” in general, the conformational properties of the ligands have also been taken into strong consideration by LBDD methods. One main example is the generation of “3D pharmacophores”, which take into account both the atomic and the conformational features of the molecules to build proper “3D-QSAR” models [31].

#### Quantitative Structure–Activity Relationship (QSAR) Modeling and Cheminformatics

As already mentioned, one of the earliest historical needs for medicinal chemists was to be able to correlate molecular modification with biological activity data. In this perspective, the first design efforts were focused only on ligand small molecules, trying to properly tune their properties simply by following the results of experiments in a target-agnostic way. This specific field of research was the so-called “quantitative structure–activity relationship” modeling, much more commonly called by its acronym “QSAR” [32]. In the second half of the last century, this strategy was known for great methodological advances, which led to its increasing application in the drug design scenario [33,34,35]. When the exploitation of computational approaches and the tools related to them started to spread, these methods were coupled with a series of other techniques in the already known and greater concept of “cheminformatics”. Indeed, this term was defined by Gasteiger and Engel as “the application of informatics methods to solve chemical problems” [36]. This field has greatly expanded over the years [37], passing from the earliest simple data analysis methodologies applicable to chemical data to the most actual implementation of cheminformatics suites and packages in widely used programming languages. In this respect, very relevant is the creation of RDKit [38], a famous and versatile cheminformatics package for Python, which is in continuous development and whose application is well documented in the scientific literature [39,40,41]. Some of the many tasks that are executable with RDKit are molecular clustering, substructure search, compound fragmentation, chemical reaction handling, shape and structural similarity analysis [42], etc.

With the development of molecular modeling, more and more relevance has been given to the three-dimensional representation of chemical entities, which is now routinely analyzed together with the more classical two-dimensional depiction [43,44]. Indeed, the modern cheminformatics tools (RDKit included) have implemented different approaches for conformer generation and prioritization, given the great importance this aspect has both in chemical research and, even more so, in drug design [45].

SBDD methods have spread exponentially in the pharmaceutical scenario; nevertheless, cheminformatics and 3D-QSAR are still widely used, as many recent papers have assessed [46,47]. Moreover, these tools and methods have been coupled with actual machine learning approaches, resulting in algorithms able to autonomously detect structural patterns in chemical data, as well as automatically create novel QSAR models [48].

### 2.2. Structure-Based Drug Design (SBDD)

**Figure 2:**
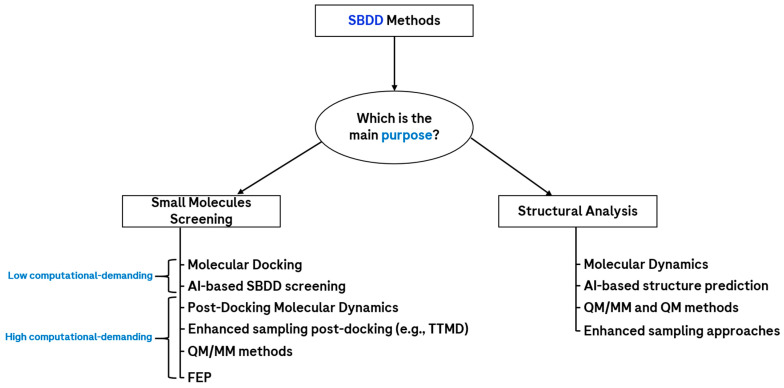
Scheme showing the main SBDD approaches, classified on the basis of their main purpose. If the main goal is to screen small molecules against a desired biological target, another important factor to consider is the computational power available, which allows roughly discriminating the techniques on the basis of the number of molecules/day screened with the same computational infrastructure, which of course plays a huge role in this perspective. Abbreviations: TTMD = thermal titration molecular dynamics; AI = artificial intelligence; QM/MM = quantum mechanics/molecular mechanics; FEP = free-energy perturbation.

With the exponential increase in the availability of three-dimensional structures of proteins and nucleic acids, which started roughly in the 2000s, the trend in the methodologies in computational drug design moved toward other techniques, which could also take into account the three-dimensional interactive features of the molecules with respect to the target. Indeed, the prior knowledge of the biological entity of interest conferred a huge advantage to the scientists, which could develop novel chemical species on the basis of its binding site characteristics. All methods based on this kind of data fall into the family of “structure-based drug design” (SBDD) [49], which are by far the most used approaches in computational drug discovery. Moreover, while some complex membrane protein structures were not considered feasible to determine experimentally 20 years ago, the modern technology of cryo-EM allowed the reliable resolution of some of those complex systems [18,50], further extending the applicability domain of SBDD. An overview of the main SBDD methods available today is reported in Figure 2.

#### 2.2.1. Molecular Docking 

Maybe the most exploited technique in modern computer-aided drug design, molecular docking, consists of the determination of the best conformation with which a molecule binds to another to form a stable complex. The name of the technique comes from the very first program which exploited it, which was called “DOCK”, proposed by Kuntz et al. in 1982 [51]. In a pharmaceutical context, these methods are extensively used to screen millions or billions of small molecules against a biological target of interest (e.g., a protein or a nucleic acid). It is important to mention that molecular docking requires prior knowledge of the binding site location on the target. A molecular docking algorithm consists of two main parts: the conformational search algorithm and the scoring function [52]. The first has the purpose to search the conformational space of the ligand considered to find a state that fits within the binding site, while the second ranks the different conformations to prioritize the most reliable [53]. Scoring functions operate on the basis of equations that take into account the conformational strain, the electrostatics, and the steric hindrance of the ligand in its bound state. Three main types of scoring functions are available today, force field-based scoring functions, empirical scoring functions, and knowledge-based scoring functions. In the first, the energy of the system is evaluated using a force field [54]. On the other hand, empirical scoring functions consist of different terms representing different intermolecular interactions, where each term is modeled using experimental values for the interaction related to it. Specifically, empirical scoring functions are based on three main pillars: descriptors for the binding event, a database of ligand–target complexes with associated experimental activity data, and an algorithm establishing a relationship between the binding descriptors and the experimental affinity [55]. The top-ranked poses by these functions are those closest to the experimental values of reference. Lastly, knowledge-based scoring functions rely on statistical analyses of the most observed interactions between a specific ligand’s atom type and a particular protein’s atom type. These functions are developed by extracting structural information from high-quality X-Ray databases (usually the Protein Data Bank and Cambridge Structural Database), and then transforming atom pair preferences into distance-dependent pairwise potentials using the Boltzmann law [56]. The top-ranked poses are those more similar to what is statistically retrievable in the experimental databases [57].

Hundreds of different molecular docking algorithms exist today, each coupling different search algorithms and scoring functions. Even if they are different, they can be grouped into different families on the basis of how they operate to find the “bound” state of the ligand. Some famous families are represented by genetic algorithms (among which the program GOLD is one of the most known and used [58]), systematic algorithms (such as the program Glide, developed and distributed by the company Schrödinger [59]), and ant colony optimization algorithms (such as the program PLANTS [60,61,62]. Some docking programs have also gained popularity due to both the robustness of their algorithms and the choice of their creators to freely distribute the software without restrictions. This is the case of AutoDock [63] (latest version 4.2.6, updated 14 April 2023) and AutoDock VINA [64] (latest version 1.2.0, updated 14 April 2023), which were both developed by the Scripps Research Institute. Both these programs have been successfully applied in small molecule research, as assessed in the literature [65].

Another classification for molecular docking is related to the degrees of freedom considered in the calculation. Indeed, in “rigid docking”, both the ligand and the protein are kept rigid. The “flexible ligand docking” approach, on the other hand, allows the ligand to explore different conformational states, keeping the target rigid [66]. Then, the “semi-flexible” or “induced fit” approach consists of taking into account the conformational spaces of both the ligand and the binding site residues [67], avoiding the scenario that small clashes with a rigid side chain could impair the selection of reasonable docking poses. In the last method, which is “ensemble” docking, molecular docking is executed against an ensemble of protein conformations, usually coming from molecular dynamics simulations. In this way, the full flexibility of the protein can be indirectly taken into account [68]. A less computationally demanding strategy to obtain conformational ensembles relies on the exploitation of multiple experimentally resolved structures of the target of interest, captured in different conformational states.

As already mentioned, molecular docking is extensively used in the early phases of drug discovery, from hit identification up to lead optimization [69]. It is applied both to identify novel chemical candidates for pharmacological testing and to help rationalize experimental data to a molecular level. Nevertheless, molecular docking is essentially a “static” approach, which considers only the final state of the ligand–target system and is performed mainly in a vacuum [70]. Indeed, even if water molecules are included in the docking calculations, this information has to be given explicitly [71], and this requires structural information coupled with molecular dynamics data. As a result of all of this, the main problem of molecular docking is the high false-positive ratio of the compounds selected by the scoring functions [72], which has induced CADD scientists to investigate ways to filter the poses produced by molecular docking algorithms using other approaches. Today, many of these methods are available. One of the simplest is called “consensus docking”, which relies on the principle that, by exploiting different docking programs based on different algorithms, the molecules prioritized by them will have a lower probability of being false positives [73,74]. The success of such an approach has been extensively demonstrated in the literature [75]. Other methods, called “post-docking” techniques, further filter the poses produced by molecular docking on the basis of certain molecular features [76]. One example of the first case is the implementation of a three-dimensional pharmacophore, in which the most relevant 3D features for the interaction with the target are embedded. In this case, only the docking poses which can respect these boundaries are kept, discarding the others [77]. Regarding the second kind of technique, one example is the implementation of molecular dynamics as a “post-docking” approach [78]. The poses in which the molecules can keep the interaction pattern with the protein for a longer simulation time are referred to as more “kinetically stable”, while the others are deprioritized. A novel, simple, and effective technique exploiting this approach, as well as implementing a temperature increase with simulation time, was recently developed, known as “thermal titration molecular dynamics” (TTMD) [79,80], which is further discussed in the next section.

#### 2.2.2. Molecular Dynamics

**Figure 3:**
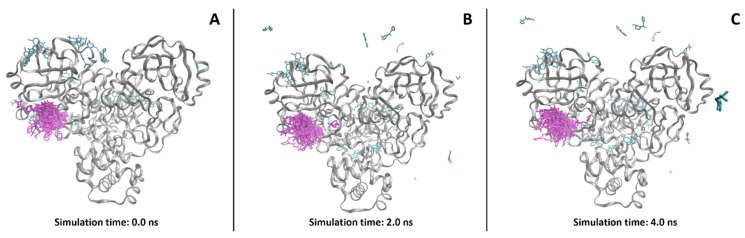
Example of the evolution of a system (from **A**–**C**) using molecular dynamics simulations, taken from a recent study published by our lab [89]. As can be seen, each of the SARS-CoV-2 M^pro^ crystallographic ligands starts from a defined position (the crystallographic one) at the beginning of the simulation. Then, after the MD is started, the molecules outside the catalytic pocket (colored in cyan), which are tendentially more exposed to the solvent, are more prone to lose the initial conformation and, eventually, the binding site itself. In contrast, the compounds which are crystallized in the catalytic pocket (depicted in magenta) are more prone to keep their initial position during the simulation, being more strongly bound to the protein.

Molecular dynamics (MD) is a computational technique used to investigate the dynamic behavior of a chemical and/or biological entity over time [81]. The method consists of the iterative resolution of Newton’s equations of motion to continuously predict the atomic positions of the molecules considered with respect to each other during the simulation time [82]. Molecular dynamics is used for various purposes in drug discovery, from the simple dynamic evaluation of a system to the mechanistic understanding of a molecular process [83], or as a well-known post-docking filtration system [84] (Figure 3). The main advantage of this method is that, in contrast to molecular docking, the system considered is free to move and, thus, more “realistic” if we consider the environment in which the real biochemical processes will happen. Moreover, molecular dynamics can be executed using explicit solvent models (e.g., TIP3P [85]), in which the contribution of each single water molecule is taken into account. This allows a better understanding of the role of water molecules in target stabilization, as well as in ligand–target recognition. The first and main drawback of molecular dynamics is certainly related to the computational power required for its implementation in the pipeline. Indeed, depending on the simulation time and on the dimensions of the system to evaluate, MD can require tens to thousands of time/molecule more than molecular docking; for this reason, its application is usually limited to a lower number of compounds (e.g., in the post-docking approaches). Furthermore, MD simulations rely on molecular mechanics and force fields in order to extrapolate atomic velocities with time; if this exponentially increases the speed of the simulations with respect to the quantum-based methods, it also carries several approximations that have to be known and taken into account by CADD scientists [86]. First, polarizability is not conceived in force-field-based MD simulations, whereby every molecule of the system has fixed bond lengths and partial charges, and no bond can be created or broken (except for QM/MM methods, which includes a focused region calculated at the QM level [87]). In recent years, the continuous increase in computational power of modern hardware architecture is allowing quantum-mechanical calculations to be more and more affordable, possibly leading to a new era in computational drug discovery [88].

##### Enhanced Sampling Methods in Molecular Dynamics

One of the major issues in simulating a molecular event is represented by the differences in timescales between the biological and the virtual environments. Indeed, episodes such as ligand unbinding may require hundreds or even thousands of nanoseconds of simulation. Ligand binding is usually even more demanding if unbiased, and events such as major conformational changes of biological entities would rarely take less than several microseconds of simulations to be sampled [90,91]. These ranges in timescales come mainly from the fact that the event to be captured is considered to be “rare” and, by definition, harder to sample [92]. In the years, many different methods have been introduced and developed to increase this sampling, favored by the introduction of some kinds of biases in the simulated system [93]. Some remarkable examples are steered MD [94], scaled MD [95], replica exchange MD [96], metadynamics approaches [97], and Gaussian accelerated molecular dynamics [98]. The first of these techniques, which is mainly used for ligand unbinding sampling, relies on the introduction of a coordinate-defined force that “guides” the ligand away from its initial placement in the binding site. The reconstruction of the free-energy profile is then possible from the Jarzynski equality, but tendentially only if the forces introduced are limited in magnitude. Scaled MD, on the other hand, involves the introduction of a scaling factor for the regulation of the potential energy of the solutes’ degrees of freedom in the simulation. Replica exchange MD is based on the swap of atomic positions between parallel simulations carried out at different temperatures, by employing independent Monte Carlo random walks. This allows a fairly augmented sampling of the system’s events. Lastly, metadynamics relies on the iterative “filling” of the potential energy in the simulation with a series of Gaussian curves, to force the system to explore different minima and, hence, improve the sampling of “rare” events. Lastly, the Gaussian accelerated molecular dynamics (GaMD) approach consists of enhancing the conformational sampling of molecules by smoothening the potential energy surface through the addition of a harmonic boost potential that follows Gaussian distribution. Recently, Yu et al. applied multiple-replica Gaussian accelerated molecular dynamics (MR-GaMD) for the analysis of the mutation-induced conformational changes in the GTPase NRAS [99]. A very similar approach was implemented in a very recent study by Chen et al., in which GaMD was exploited for the investigation of S-adenosyl-l-methionine (SAM)-responsive riboswitches [100]. Together with these methods, some other enhanced sampling techniques have been developed in the years, eventually allowing a proficient sampling of the desired biological event with lower to no bias introduction in the system. This is the case of supervised molecular dynamics (SuMD) [101] and thermal titration molecular dynamics (TTMD) [79], which are discussed in the next section.

##### Molecular Dynamics as a Post-Docking Approach

As already mentioned, one of the main applications of molecular dynamics in the drug discovery pipeline consists of its exploitation in the discrimination of molecules after a molecular docking run. Indeed, while molecular docking gives only a static representation of the binding event, focusing on the final state only, MD is able to evaluate the dynamic stability of such conformation in the binding site. In post-docking MD, the poses resulting from the docking calculations are then used as the starting point for molecular dynamics, which samples diverse short simulations (usually very few nanoseconds long) for the complex considered [102]. The parameters usually tackled from this perspective are of a geometric kind, such as the root-mean-square deviation (RMSD) and the root-mean-square fluctuation (RMSF) of atomic positions. While the first describes how much a molecular entity (e.g., the ligand) displaces from its initial position during the simulation, the second quantifies the magnitude of the displacement from the most frequent position, indicating the “fluctuation” of the entity itself. Even if such parameters are easy to calculate and compare, they often offer a poor description of the binding quality. Indeed, small changes in RMSD can bring the ligand to completely lose its interaction pattern during the simulation, while higher deviations in RMSD could be due to some flexible moieties that are exposed to the solvent and, thus, able to freely fluctuate in it. To overcome these limitations, other metrics should be considered to evaluate MD-based post-docking replicas. One of the examples of this is the tackling of protein–ligand interaction fingerprints (PLIFs), which can be compared for all the MD frames, allowing for evaluation of the effective quality of the interactions described by the molecular docking poses [53]. In this perspective, the molecule with the most proficient interaction patterns keeps the PLIFs during the MD simulations, while more weakly interacting compounds tendentially lose the contacts which stabilize their bound conformation.

##### Free-Energy Perturbation (FEP) and Thermodynamic Integration (TI)

In recent years, the increase in hardware performance (especially looking at the power of graphics processing units—GPUs), together with the advances in parallel computing, has allowed the spreading of the application of free-energy perturbation (FEP) methodology [103]. Specifically, this technique allows, though the exploitation of the thermodynamic free energy cycle, to extrapolate of the relative binding free energy of a series of co-generic ligands (the changes among the tested small molecules should be restricted to <10 atoms) [104]. This approach is gaining exponentially increasing interest in the world of drug design, with applications for targets ranging from protein kinases [105] to viral proteases [106] and GPCRs [107]. Specifically, FEP can be discussed in two main terms, which are absolute binding free energy (ABFE) and relative binding free energy (RBFE). The first refers to the calculation of the binding energy of a solvated ligand to a target, while the second regards the relative free energy of binding between two ligands and a target [108,109].

The advances in the predictive accuracy of these methods are mainly attributable to the improved force fields (e.g., the latest releases of the OPLS force field [110], implemented in the FEP+ application from Schrödinger [111]) and novel advances in sampling algorithms [112]. Nevertheless, it is crucial to remember that much importance has to be given to the system setup before performing an FEP calculation, to allow it to return reliable ∆∆G values. Indeed, the positions of the binding site waters should be accurately defined, the co-generic ligands should be docked in a way that their bound conformation is almost superimposable (at least for the common scaffold), and some MD simulations should be executed on the starting ligand, to ensure that its binding mode is stable [113].

With FEP, it is also feasible to understand the importance that each molecular portion has for binding, weighting it in quantitative terms according to the binding free energy. In its latest implementation, uncertainties <1 kcal/mol were reached, comparable to the experimental error associated with the measurement of the actual values [110]. Free-energy perturbation methods have already been applied successfully to different scenarios in drug discovery [114,115].

Thermodynamic integration (TI), on the other hand, can be referred to as a method to compare the difference in free energy between two given states. These states are characterized by different potential energies, and these have different dependences on the spatial coordinates of the entities involved in the simulated system [116,117]. Unlike FEP, which relies strongly on MD or Monte Carlo simulations, TI calculates the difference in free energy between these states by integrating over ensemble-averaged enthalpy along an alchemical path. 

As in the case of FEP, the integration step is dictated by the coupling parameter λ; in TI, the potential energy of the first state is associated with λ = 0, while the final state has λ = 1 [118]. TI has successfully been applied to molecular biology [119] and drug discovery [120,121,122] for the prediction of the binding free energy between chemical and biological entities, and its implementations come often together with other techniques, resulting in approaches such as independent trajectories thermodynamic integration (IT-TI, in which the “independent trajectories” term is similar to a replica-exchange method) [123], density field thermodynamic integration (DFTI) [124], and umbrella integration (UI) [125].

With both FEP and TI, the main limitations are related to the fact that the high reliability of alchemical methods, other than requiring a deep prior structural and functional knowledge of the simulated system, is unequivocally bound to the molecular similarities of the compounds considered, which all have to belong to the same chemical series.

##### Thermal Titration Molecular Dynamics (TTMD)

**Figure 4:**
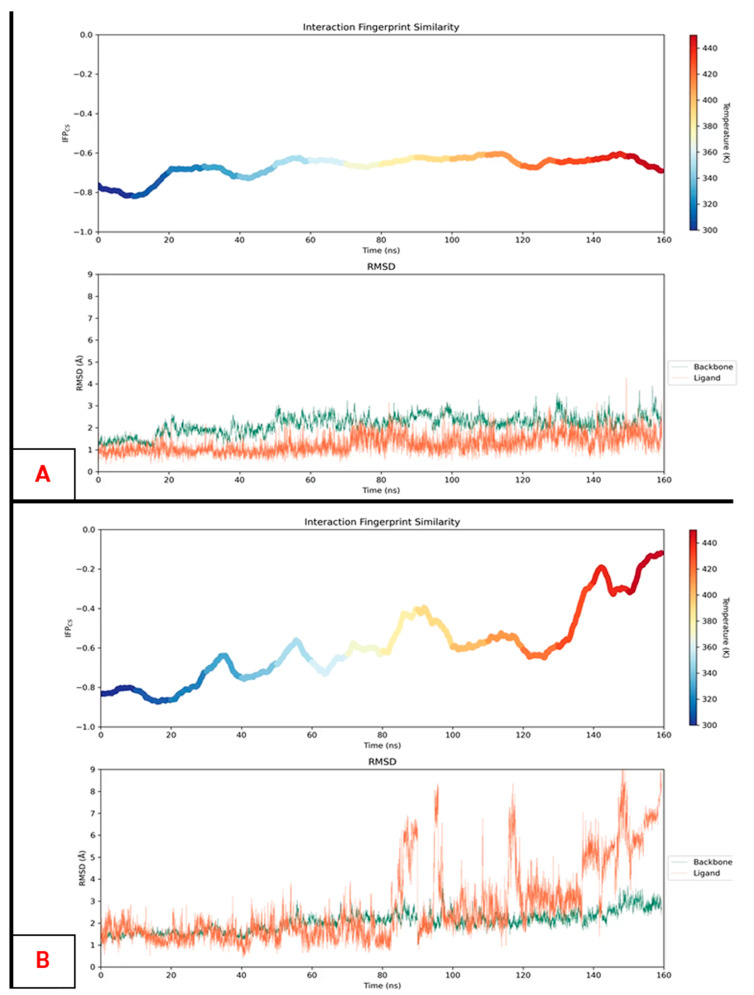
Examples of two TTMD profiles. On the upper side of each panel, is it possible to depict the IFP_CS_ score change during the simulation, where higher scores represent a loss in the initial protein–ligand interaction fingerprints and, consequently, a loss of the binding mode. The lower part of each panel shows the root-mean-square deviations (RMSDs) of the protein backbones (in green) and the ligand (in orange) against the simulation time. These last plots allow assessing that the increase in temperature does not affect the protein folding in a relevant fashion. **Panel A** represents the results of the TTMD simulation for the ligand PF670462 in the pocket of casein kinase 1δ (CK1δ), starting from the bound conformation of the crystal 3UZP [127], while **panel B** depicts the results of the TTMD experiment for an epiblastin A brominated derivative bound to CK1δ, coming from the crystal with PDB code 5IH6 [128]. As can be observed, the nanomolar ligand PF670462 keeps its protein–ligand interaction fingerprint during the simulation, while the micromolar epiblastin A derivative progressively loses the initial binding mode, assessing the instability of its contact with the protein.

One of the limitations of MD-based post-docking methods is related to the fact that, in many situations, some nanoseconds of simulation are not enough to discriminate potentially good from potentially weak binders [126]. Indeed, in many scenarios, both kinds of ligands keep low RMSD and RMSF values through the MD replicas, and their PLIFs are also equally maintained. To overcome this limitation, our group recently proposed a new method, named “thermal titration molecular dynamics” (TTMD), which proficiently takes advantage of the “concept of temperature” in molecular mechanics and molecular dynamics to classify ligands on the basis of their on-target affinity [79]. Indeed, in classic MD, the temperature is simply a value used to scale the atomic velocities with time and is not related to the “real-life” concept of temperature, which heavily influences all biochemical processes. This allows MD simulations to be performed at temperatures above 350 K or even 400 K without seeing any unfolding event taking place; in the experimental setup, these values would totally compromise the experiment. Accordingly, the TTMD method consists, starting from a protein–ligand complex, of executing MD replicas in which, for every TTMD step (T_i_), the temperature of the system is increased to a certain defined number of degrees. While this process takes place, the PLIFs are monitored, along with the RMSD of the protein backbone (which can be used as a metric to effectively assess the protein integrity during simulations). The TTMD experiment may end in two different ways: when the PLIFs are lost, or after a user-defined simulation time. Examples of the results of two TTMD experiments are given in Figure 4.

**Figure 5:**
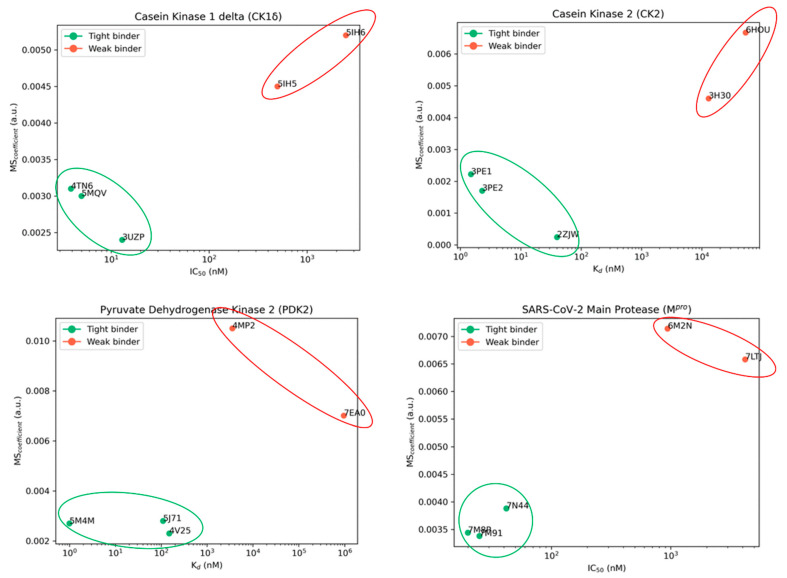
The results of the first published application of thermal titration molecular dynamics (TTMD). As can be seen, in all case studies considered, the method was able to efficiently discriminate nanomolar ligands (indicated with the green dots and highlighted with the green circles) from micro- and millimolar ones (depicted with red dots and circled in red). The MS coefficient, which was used for the classification, depends on the ability of each molecule to preserve its protein–ligand interaction fingerprints (PLIFs) during the TTMD experiment (a more detailed and mathematical explanation of its derivation was reported in [79]).

In its first implementation, this method was applied to four different case studies (CK1δ, CK2, PDK2, and SARS-CoV-2 M^pro^), taking five different crystal structures each. The potencies of the ligands in such complexes were known experimentally. The TTMD experiment was set up with a temperature ramp from 300 K to 450 K, with an increase of 10 K every 10 ns of simulation. In all cases considered, TTMD was effectively able to clearly distinguish the potent nanomolar ligands from weaker micro- to millimolar ones (Figure 5). The potential of this simple approach was also more recently confirmed with its application in MD-based post-docking approaches, given its intrinsic advantage to overcome the eventual inability of classical MD to discriminate ligands on a reduced timescale [80].

#### 2.2.3. Supervised Molecular Dynamics

Supervised molecular dynamics (SuMD) consists of an MD approach that is designed to describe a “rare” molecular binding event on a reduced timescale [101]. In the specific case of SuMD, the event considered is the target–ligand recognition process, which would require simulations on the timescales of microseconds to be described by a classical MD simulation. This is due to the huge amount of time that the ligand would spend simply fluctuating in the free solvent, without any contact with the target and, even less, with the binding site. Indeed, classical MD is referred to as a “low-sampling” approach, because the force fields can only very partially sample the potential energy surface of the system. Usually, to overcome these limitations, computational approaches include Markov state models and the enhanced sampling techniques, as discussed previously. In the first case, the MD simulation is considered an ensemble of microstates, which are independent of one another. The algorithm then calculates the transition probability matrix, which allows deriving the probability of the system passing from one microstate to another [129]. On the other hand, enhanced sampling methods consist of the perturbation of the potential energy surface of the system, allowing an escape from local minima [130].

**Figure 6:**
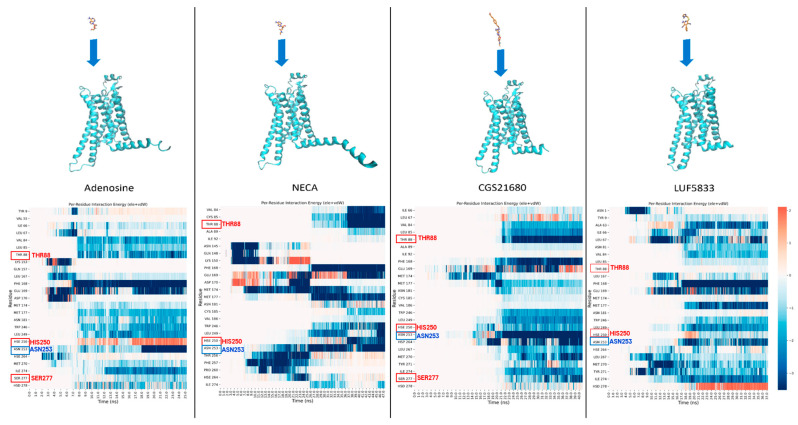
Results of the application of supervised molecular dynamics (SuMD) to the elucidation of the differences in the ligand binding paths between full agonists (adenosine, NECA, and CGS21680) and a non-ribosidic partial agonist (LUF5833) of the adenosine A_2A_R receptor. The panels in the upper part of the figure represent the initial configuration of each SuMD simulation, with the ligand placed away from the orthosteric binding site. The plots in the lower part depict the outcomes of the time-based per-residue interaction analysis, in which the summation of electrostatic and van der Waals contributions for each of the 25 most contacted protein residues is reported for each frame of the trajectories produced. In this study, SuMD highlighted the main differences between the ligand–protein recognition paths of full agonists and LUF5833, which can exert a partial agonism to A_2A_R, even if important residues such as Thr88 and Ser277 (which are labeled in the plots in red) are not recruited directly by this specific compound. An explanation of the molecular reasons behind this behavior was reported in the original publication by Bolcato et al. [133].

SuMD, on the other hand, is a technique in which no perturbation of the potential energy surface takes place. It is based on a supervision algorithm that evaluates the distance between the ligand and its binding site on the target in an iterative fashion. Specifically, the ligand is placed at a distance from the binding site in which there is no possibility of interaction at the beginning of the simulation (at least 40 Å away), and then a series of small MD simulations are sampled. At the end of each one of these, the distance between the ligand and the binding site is calculated; if the value is lower than the distance at the beginning of the small MD simulation, only the final coordinates are kept, and another MD is started from those. Otherwise, the initial coordinates are restored, and the MD simulation is repeated. When the ligand reaches a defined distance threshold (5 Å, by default), the supervision is shut down, and the simulation continues as a classical MD, allowing the ligand to relax into the binding site. In the end, the final “SuMD trajectory” is obtained by merging all small MD simulations [131]. The great advantage of this method is that it can describe an event such as the target–ligand recognition process on the nanosecond timescale rather than microseconds (typical of classical MD), accomplished without the introduction of energetic biases. This technique has already been extensively used on different targets, such as G-protein-coupled receptors (GPCRs) [132,133] (see Figure 6), proteases [134], and kinases [135], considered as ligands, small molecules, peptides [136], and aptamers [83]. The SuMD analysis allows getting a visual representation of the most relevant residues for the interaction with the target at each step of the simulation, providing very relevant information to how the approaching process influences the binding, as well as regarding the eventual “meta-binding sites” present on the target.

## 3. Conclusions and Future Perspectives

In this review, we summarized the advantages and disadvantages of some of the main computational methods applied for computer-aided drug design in the past and today, looking at their origin, rationale, application, and future perspectives. The continuous advances in both methodological development and informatics infrastructure are now motivating another push through the boundaries of drug discovery. Indeed, AI-related approaches are attracting great importance in the fields of molecular property and behavior prediction [137] wherever enabled by the amount of data. Several different fields of computational chemistry have already experienced the benefits given by artificial intelligence, with a special focus on the early discovery environment [138,139]. One very relevant example is represented by on-target and off-target effect predictions in computational toxicology [140,141,142], which is configured in the family of AI-based methods for target prediction based only on ligand chemical data. On the structure-based side, delta-learning [143], deep learning-based 3D pocket mapping [144], and AI rescoring techniques have been developed and documented in the recent years. These latest methods have gone beyond the prioritization performance offered by the classical scoring approaches [145,146].

On the other hand, quantum mechanics methods are becoming more and more feasible [147]. The application of AI for QM property prediction is already established in the CADD field [148,149], but it is possible to foresee that, with the advent of quantum computing, the massive calculation of such attributes will become routine.

This review can be helpful to scientists approaching the world of CADD and computational chemistry applied to pharmaceutical development, serving as a helpful tool for gaining an understanding of the possibilities that these strategies have in improving the success rate of drug discovery pipelines.

## Data Availability

Not applicable.
