# Correction: Bassani, D.; Moro, S. Past, Present, and Future Perspectives on Computer-Aided Drug Design Methodologies. *Molecules* 2023, *28*, 3906

**DOI:** 10.3390/molecules28135223

**Published:** 2023-07-05

**Authors:** Davide Bassani, Stefano Moro

**Affiliations:** Molecular Modeling Section (MMS), Department of Pharmaceutical and Pharmacological Sciences, University of Padova, Via Marzolo 5, 35131 Padova, Italy; davide.bassani@roche.com

In the published publication [1], there was an error regarding the affiliations for **Davide Bassani**. The affiliation **Pharmaceutical Research & Early Development, Roche Innovation Center Basel, F. Hoffmann—La Roche Ltd., 4070 Basel, Switzerland** should be removed. The correct version of the affiliation should be:

Davide Bassani and Stefano Moro * 

Molecular Modeling Section (MMS), Department of Pharmaceutical and Pharmacological Sciences, University of Padova, Via Marzolo 5, 35131 Padova, Italy; davide.bassani@roche.com

* Correspondence: stefano.moro@unipd.it

The authors state that the scientific conclusions are unaffected. This correction was approved by the Academic Editor. The original publication has also been updated.
